# Calretinin immunoreactivity in the claustrum of the rat

**DOI:** 10.3389/fnana.2014.00160

**Published:** 2015-01-20

**Authors:** Rastislav Druga, Martin Salaj, Filip Barinka, Lawrence Edelstein, Hana Kubová

**Affiliations:** ^1^Department of Anatomy, Charles University in Prague, 2nd Faculty of MedicinePrague, Czech Republic; ^2^Department of Anatomy, Charles University in Prague, 1st Faculty of MedicinePrague, Czech Republic; ^3^Department of Developmental Epileptology, Institute of Physiology, Academy of Sciences of the Czech RepublicPrague, Czech Republic; ^4^Department of Neurology, University of RegensburgRegensburg, Germany; ^5^Medimark CorporationDel Mar, CA, USA

**Keywords:** calcium-binding proteins, calretinin, claustrum, endopiriform nucleus, rat

## Abstract

The claustrum is a telencephalic structure which consists of dorsal segment adjoining the insular cortex and a ventral segment termed also endopiriform nucleus (END). The dorsal segment (claustrum) is divided into a dorsal and ventral zone, while the END is parcellated into dorsal, ventral and intermediate END. The claustrum and the END consist of glutamatergic projection neurons and GABAergic local interneurons coexpressing calcium binding proteins. Among neurons expressing calcium binding proteins the calretinin (CR)-immunoreactive interneurons exert specific functions in neuronal circuits, including disinhibition of excitatory neurons. Previous anatomical data indicate extensive and reciprocally organized claustral projections with cerebral cortex. We asked if the distribution of cells immunoreactive for CR delineates anatomical or functional subdivisions in the claustrum and in the END. Both segments of the claustrum and all subdivisions of the END contained CR immunoreactive neurons with varying distribution. The ventral zone of the claustrum exhibited weak labeling with isolated cell bodies and thin fibers and is devoid of immunoreactive puncta. Within the medial margin of the intermediate END we noted a group of strongly positive neurons. Cells immunoreactive for CR in all subdivisions of the claustrum and END were bipolar, multipolar and oval with smooth, beaded aspiny dendrites. Small number of CR-immunoreactive neurons displayed thin dendrites which enter to adjoining structures. Penetration of dendrites was reciprocal. These results show an inhomogenity over the claustrum and the END in distribution and types of CR immunoreactive neurons. The distribution of the CR-immunoreactive neurons respects the anatomical but not functional zones of the claustral complex.

## Introduction

The claustrum is a pallial/subcortical structure which, in the rat, consists of a dorsal segment (CLD), termed also insular claustrum adjoining the insular cortex, and a ventral segment also termed endopiriform nucleus (END) situated deep to piriform cortex. The CLD is further parcellated into a dorsal part of claustrum (DCl) and a ventral part of claustrum (VCl; Paxinos and Watson, 2007). Paxinos and Watson (1997) divided the END into two parts, the dorsal endopiriform nucleus (DEn) and the ventral endopiriform nucleus (VEn). In the most recent edition of their stereotaxic atlas (Paxinos and Watson, 2007) END is divided into three parts: the DEn, the VEn and the intermediate endopiriform nucleus (IEn). This recent subparcellation of END was used also in our present study. The claustrum is reciprocally and topographically connected with most if not all neocortical and allocortical areas (Edelstein and Denaro, 2004; Druga, 2014). Studies on the expression of developmental regulatory genes specific to the lateral and ventral pallial histogenetic divisions of mammalian brain indicate that CLD and END, together with the pallial part of the amygdala, may be regarded as a single entity, named the “claustroamygdaloid complex” (Medina et al., 2004).

The CLD and END consist of glutamatergic projection neurons and GABAergic local interneurons coexpressing calcium-binding proteins (CBPs) and neuropeptides in various combinations (Kowiański et al., 2009). Calcium-binding proteins are classified as “buffer proteins,” which may act as modulators of cytosolic calcium levels. The expression of CBPs is often used for revealing functionally distinct subdivisions of the CNS and for the detection of various classes of inhibitory interneurons. Calretinin (CR) is one calcium-binding protein with complex Ca2+ -binding kinetics (Schwaller, 2014). In addition to its buffer function it might also serve as a Ca2+ sensor. Further, CR is involved in the regulation of neuronal excitability and synaptic plasticity (Camp and Wijesinghe, 2009). CR-immunoreactive (CR-ir) interneurons have been shown to influence the activity of other interneurons (Gulyás et al., 1996), in some cortical areas a gating function of CR-ir interneurons has been presumed (Callaway, 2004; Barinka et al., 2012). There are some data indicating resistence of CR-ir neurons in several neurological and psychiatric disorders (Camp and Wijesinghe, 2009; Barinka and Druga, 2010; Schwaller, 2014). While the various functions of CR interneurons have been partly elucidated, its expression in tumor cells (e.g., mesothelioma, soft tissue tumors) has proven to be enigmatic (Barak et al., 2012).

Although Paxinos et al. (1999) reported that the rat claustrum is defined by the absence of CR staining, subsequent studies in the mouse have reported CR-immunoreactivity in both subdivisions of the claustrum (Real et al., 2003, 2006). In addition, Wójcik et al. (2004) reported CR-ir neurons in the dorsal claustrum of the rabbit, Reynhout and Baizer (1999) in the monkey claustrum and Rahman and Baizer (2007) in the cat claustrum. Recently, Cozzi et al. (2014) described CR-ir neurons in the claustrum of the dolphin. The objective of the present study is to define the distribution of CR-ir neurons in all subdivisions of the claustrum of the rat, while searching for patterns related to connectivity, functional characteristics and histogenetic parcellation. Specifically, we sought to analyze the distribution of dendritic arborizations of CR-ir neurons and their extensions to adjacent cortical and subcortical structures. Our results complement the mouse and guinea pig data of Real et al. (2003) and Edelstein et al. (2010).

## Materials and methods

Experiments were carried out in six adult male Wistar rats (350–400 g). All animals were cared for in accordance with the regulations and laws of the European Union (86/609/EEC) as well as NIH guidelines (Assurance No. A5820-1). Experiments were approved by the Animal Care and Use Committee of the Institute of Physiology of the Academy of Sciences of the Czech Republic. Animals were housed under standard conditions (12 h light/12 h dark cycle, 22 ± 1°C, humidity 50–60%, free access to food and water).

Rats were irreversibly anesthetized (urethan 2 g/kg i.p.) and transcardially perfused with 0.01 M phosphate-buffered saline (PBS; pH 7.4), immediately followed by 4% paraformaldehyde in 0.1 PBS. The brains were removed from the skull, postfixed in buffered 4% paraformaldehyde for 3 h and then cryoprotected via a standard sucrose gradient in PBS at 4°C. The brains were sectioned in the coronal plane at 50 μm with a cryocut Leica CM 1900. Four sets of sections through anterior—posterior extent of the claustrum were processed; three sets were used for immunocytochemistry to CBP (Parvalbumin, Calbindin, Calretinin), and a fourth was stained with cresyl violet. Intervals between sections immunostained for CR was 200 μm. Free-floating sections were incubated in 0.15% hydrogen peroxide in PBS for 10 min, rinsed with PBS five times, permeabilised with 0.3% Triton-X100 for 5 min and then incubated with a blocking solution containing 2% horse serum (Vector Laboratories, Burlinghame, CA, USA) for 1 h at room temperature. The sections were then incubated with the primary antibody (mouse anti-CR monoclonal, Millipore, dilution 1:8000, catalogue No. MAB1568) in PBS containing 1.5% normal horse serum and 0.1% Triton-X100 for 48 h at 4°C and then rinsed five times in PBS. Thereafter sections were then incubated for 1 h at room temperature with secondary antibody (biotinylated anti-mouse made in horse, Vector, dilution 1 : 50) in PBS containing 1.5% normal horse serum. Lastly, a standard ABC kit was used (Sigma-Aldrich). The peroxidase reaction was carried-out by using as the chromogen DAB (Sigma-Aldrich).

No immunostaining was seen in the control sections where incubation with primary antibody was omitted.

In each animal 25–28 sections immunostained for CR were analyzed.

Measurements of neuronal diameter and cross-sectional area were performed under a 40 × objective by means of the imaging acquisition application cell^F^ (Olympus). The average values of these parameters along with standard error of mean (S.E.M.) are provided. In each animal (6 animals), 20 neurons in each subdivision were evaluated. Hence, together 120 neurons in each subdivision were analyzed (alltogether, 600 neurons were analyzed). Selected CR-ir neurons with extensive dendritic trees were drawn using a camera lucida attachment. Light-microscopic images were photographed with an Olympus Bx51 microscope equipped with an Olympus digital camera DP 72. Digital images were loaded into Adobe Photoshop.

### Densitometric analysis

Densitometric analysis of CR immunopositivity in areas DCl, VCl, DEn, VEn and IEn was performed in seven coronal sections (2.1 mm, 1.2 mm, 0.2 mm, −0.8 mm, −1.8 mm, −2.8 mm and −3.8 mm AP relative to bregma (Paxinos and Watson, 2007); sections A, B, C, D, E, F and G in Tab. 2) evenly spaced along the rostrocaudal axis. The pictures were captured using an image analyzing software (QuickPHOTOMICRO 2.3, Promicra) and digital kamera (OlympusDP 72) attached to the microscope Olympus BX51. 4 × objective was used. To avoid differences in light intensity of the captured images, all images were captured at the same light intensity in the microscope (6 on the microscope scale). The images were converted to grayscale. Densitometric analysis was performed using software Densita, MBF Bioscience (MicroBrightField, Inc.), an integrated system for quantitative analysis of optical density in biological tissue. To get true scaling of the acquired images we calibrated grid using scale bar image acquired under the same conditions as the immunohistology images. Then we load the calibration strip image with solid blocks of defined grayscale values to capture calibration luminance values and to calculate related relative optical density (ROD) values. The ROD was calculated using formula:

$$ROD_{AVG}=\frac{1}{N}\sum_{i=0}^{n}\log_{10}\left(\frac{F}{P_{i}}\right),$$

where *F* is the background gray level (*F* = 255), Pi is the pixel gray level and N is the number of the pixels. A lower ROD value means that more light is coming through the tissue and a higher value means more light is blocked in the tissue and therefore not detected. The linear (first order) regression line was fitted to approximate the data. Using the regression line we calculated ROD for each studied area and section. To eliminate the possible biasing influence of slightly different intensity of immunopositivity between different sections and animals, the ROD were corrected dividing the values measured in individual areas under study by the values from the corpus callosum on the corresponding sections. This corrected staining index (corrected relative optical density, cROD) allowed comparison among different sections and animals. The corpus callosum was selected as the reference structure because it is weakly immunolabeled by antibodies to CR and has well-defined borders.

### Statistical analysis

Analysis of variance (ANOVA) followed by Tukey-Kramer multiple comparison test was used for the comparison of corrected relative optical densities as well as for the differences in morphometric parameters of neuronal somata between subdivisions of claustral complex. Probability (*p*) values < 0.05 were considered significant.

## Results

### Nissl staining

In the rat the claustrum (CLD) is located underneath the insular cortex and shows elliptic or drop-like shape. Its major axis is oriented from dorsolateral to ventromedial. Anteroposteriorly claustrum extends from rostral pole of the striatum to posterior part of thalamus (from AP 2.5 to AP −1.9). The END exhibits elliptic configuration its major axis is vertically oriented and paralel with external capsule Anteroposterior extent of the DEn was from AP 2.5 to AP −4.3 while the anteroposterior extent of the IEn and VEn was shorter 3.1 mm and 2.2 mm.

**Figure 1:**
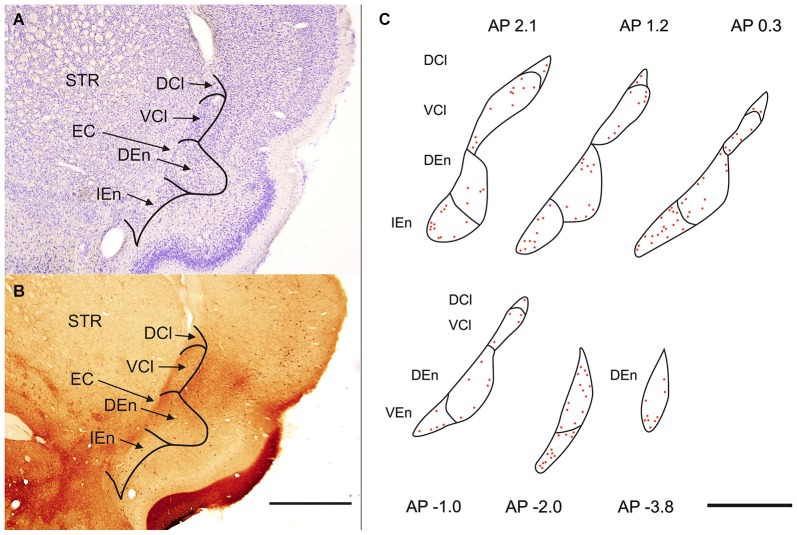
**(A)** Transverse section of rat hemisphere showing claustrum and the endopiriform nucleus (END) stained with cresyl violet. **(B)** Transverse section of rat hemisphere showing the calretinin (CR) immunoreactivity in the dorsal claustrum and in the END. DCl—dorsal part of the claustrum. An oval central region with a weakly stained neuropil and small number of CR-ir neurons corresponds with the ventral part of the claustrum (VCl). DEn—dorsal endopiriform nucleus, IEn—intermediate endopiriform nucleus. EC—external capsule, STR—striatum. Scale 1000 μm. **(C)** Distribution of CR-ir neurons is shown on camera lucida drawings. Abbreviations as in **(A)** and **(B)**. Scale 1000 μm.

Polymorph, small and medium sized round, eliptic and multipolar cells can be seen in the rat’s claustrum and in the END. The cells are tightly grouped in the VCl, while they are less densely packed in the DCl (Figure 1A). In the END its dorsal part (DEn) exhibits lower density of neurons and difference in density enables to demarcate it against the VCl. For the IEn is characterisic lower density of cells and presence of larger and more intensely stained perikarya. In comparison with the DEn the cells within the VEn are less densely packed and less intensely stained.

### Calretinin immunoreactivity

#### Claustrum

Sections processed for CR-ir exhibited densely stained cells against a backround of stained fibers and puncta. CR-ir neurons were present in all subdivisions of the claustrum as well as in END (Figures 1B,C).

**Figure 2:**
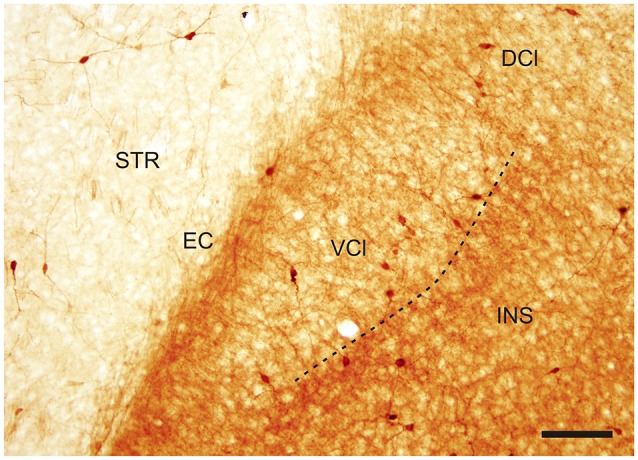
**Photomicrograph demonstrating CR-ir in the DCl and in the VCl**. Dashed line indicates boundary between insular cortex and VCl. DCl—dorsal part of the claustrum, EC—external capsule, INS—insuluar cortex, STR—striatum, VCl—ventral part of the claustrum. Scale 100 μm.

In the CLD of the rat, two zones are evident which differ relative to the CR-immunoreactivity of their neuropil (Figure 1B). One zone, which corresponds to the central part of the VCl, exhibited weak CR-immunoreactivity. This “pale nucleus” contains isolated thin fibers oriented in various directions and is practically devoid of CR-ir puncta (Figure 2). It was also seen to contain isolated CR-ir cell bodies primarily in its periphery (7–14 per section). A second zone, which corresponds partly to the DCl of Paxinos and Watson (2007), surrounds the VCl in its dorsal, medial and basal aspects with a rim of moderately CR-ir neuropil and fibers and contained small number of CR-ir neurons (2–6 per section). A neighboring zone which medially delineates the VCl and DCl from the margins of the external capsule was seen to have numerous moderately stained CR-ir puncta, perikarya and thin fibers. Lateral to the VCl and DCl, a band of moderate-to-strong CR-immunoreactivity was seen corresponding to the infragranular layers of the insular cortex. (Figures 1B, 2). Although CR-ir neurons were distributed in both zones of the CLD, they were most prevalent at VCl and in deep layers of the insular field (GI, DI, AID, AIV; see Paxinos and Watson (2007).

#### The endopiriform nucleus

**Figure 3:**
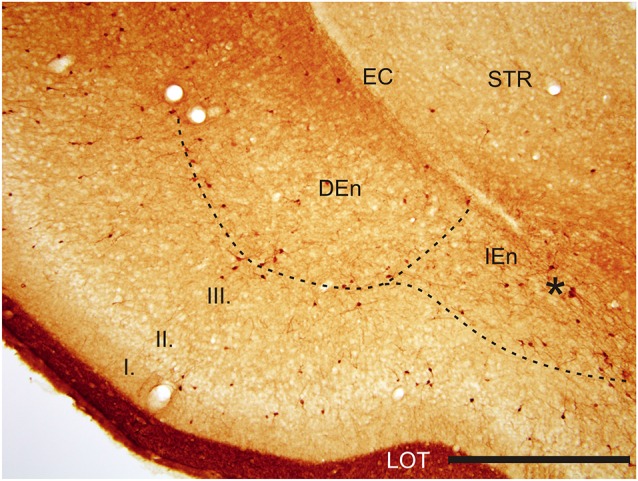
**Photomicrograph showing CR-ir in the DEn and in the IEn**. Dashed line indicates boundary between piriform cortex and DEn and IEn. I–III—layers of the piriform cortex, EC—external capsule, LOT—lateral olfactory tract, STR—striatum. *—cluster of strongly CR-ir neurons within the medial margin of the IEn. Scale 500 μm.

**Figure 4:**
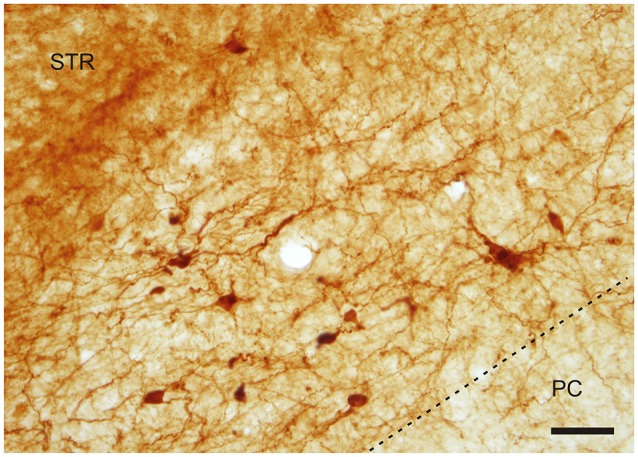
**A cluster of CR-ir neurons in the IEn**. Dashed line indicates the boundary between the IEn and the third layer of the piriform cortex (PC). Scale 50 μm.

With regard to CR-immunoreactivity in the DEn and IEn, both displayed weak-to-moderate labeling of neuropil containing a relatively small number of thin positive fibers and puncta. In the DEn, few scattered strongly positive neurons were noted (4–9 per section) (Figure 3). The medial border of DEn and IEn was indentifed by a thin rim of strongly positive neuropil which was seen to be continuous with the moderate-to-strongly positive medial border of the CLD. Ventral to the DEn and caudal to stereotaxic coordinate AP 0.5 within the medial margin of the IEn, we noted a cluster of strongly CR-ir neurons embedded within weakly or moderately stained neuropil, discernible throughout the caudal extent of the nucleus (Figure 3). Majority of neurons within this cluster exhibited medium sized or large multipolar morphology (up to 30 μm) and emerging dendrites were thicker than in other types of CR-ir neurons (Figure 4). The IEn contained 8–15 CR-ir neurons per section. As seen with the DEn and IEn, the VEn displayed moderately immunoreactive neuropil with a relatively small number of positive neurons.

#### Morphology of CR-ir neurons

With regard to generally observed somatodendritic patterns in the rat claustrum, CR-ir multipolar, oval and fusiform densely stained neurons were evident in all subdivisions. The multipolar neurons displayed smooth and occasionally beaded radially-oriented dendrites, while oval and fusiform cells typically exhibited smooth bipolar dendrites. Dendrites of all of the examined CR-ir neurons were seen to be of the aspiny type. Proximal segments of some dendrites exhibited undulated appearance. The larger diameter of positive DCl and VCl neurons averaged 13.5 ± 0.3 μm (±S.E.M.) and 14.0 ± 0.2 μm respectively, with a cross-sectional area of 73.7 ± 2.0 μm^2^ and 80.7 ± 2.2 μm^2^ respectively. The average diameter of CR-ir DEn neurons was 14.5 ± 0.2 μm, with a cross-sectional area of 85.1 ± 1.9 μm^2^. The average diameter of CR-ir neurons within the IEn was 15.6 ± 0.3 μm, with cross sectional area of 104.2 ± 2.9 μm^2^. Neurons in the VEn were bigger with average larger diameter 16.9 ± 0.4 μm amd cross-sectional area 112.3 ± 3.8 μm^2^. In the case of the average diameter, the differences DCl-IEn, DCl-Ven, VCl-IEn, VCl-VEn, DEn-IEn as well as DEn-Ven reached the statistical significance (*p* < 0.05). With exception of IEn-VEn, DCl-VCl and VCl-DEn, all interareal comparisons of cross-sectional area of neuronal perikaryon showed statistically significant differences between studied claustral subdivisions (*p* < 0.05).

**Figure 5:**
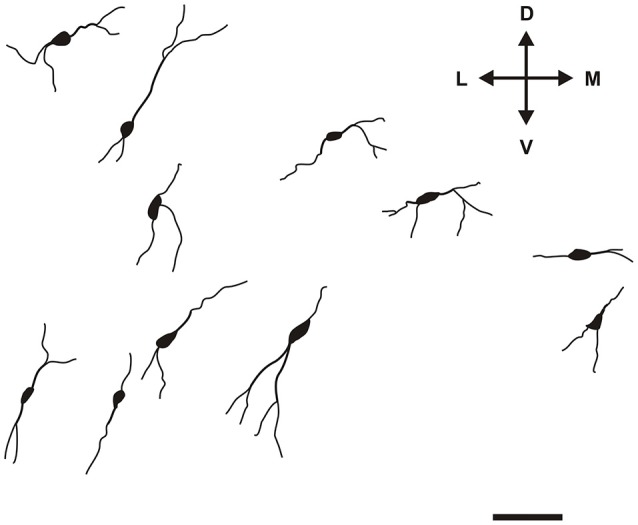
**Camera lucida drawings of various types of CR-ir neurons in the DCl**. Scale 50 μm. D, L, V, M—dorsal, lateral, ventral and medial direction.

**Figure 6:**
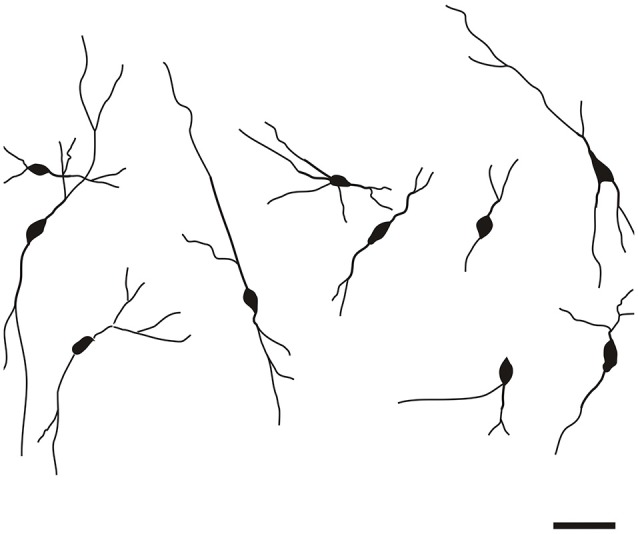
**Camera lucida drawings of CR-ir neurons in the VCl**. Scale 50 μm.

Specific to the morphology of the DCl, oval and fusiform cell bodies were most prevalent, with vertically-oriented bipolar dendrites (with the occasional horizontally-oriented type). Sparsely-branched dendrites were seen to extend 100–150 μm from the cell body (Figure 5). As for the VCl, oval and multipolar cell bodies were most commonly seen, fusiform cell bodies were observed less frequently (Figure 6). Bipolar cells were noted in close proximity to its border with the external capsule.

**Figure 7:**
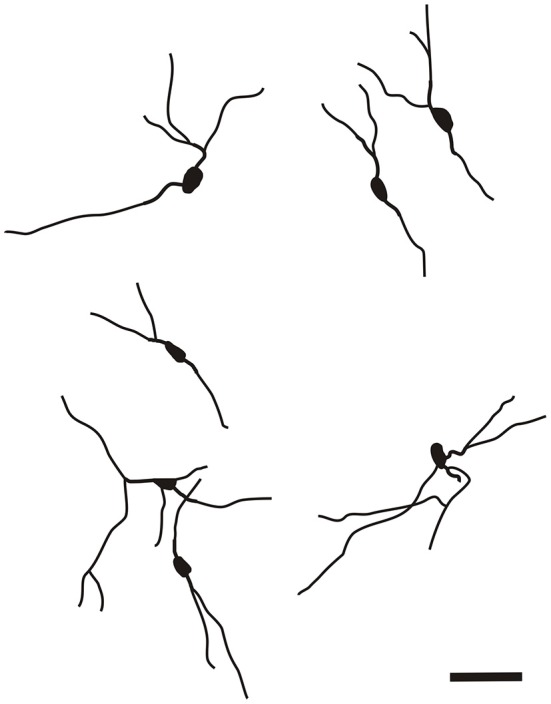
**Camera lucida drawings of CR-ir neurons in the DEn**. Scale 50 μm.

The CR-ir neurons in the DEn exhibited oval, fusiform (spindle) or multipolar perikarya with slight prevalence of oval perikarya (Figure 7).

The CR-ir neurons within the IEn were comprised of a combination of multipolar, triangular and oval perikarya with thicker primary dendrites (Figure 4).

In the VEn oval and spindle CR-ir neurons prevailed.

Very small number of CR-ir spindle neurons with bipolar morphology and long dendrites were located within the external capsule. Terminal segments of their dendrites leave the external capsule and enter the dorsal claustrum or marginal part of the striatum. In addition to vertically oriented CR-ir fibers, the external capsule contained small number of obliquelly or horizontally passing thin fibers.

**Figure 8:**
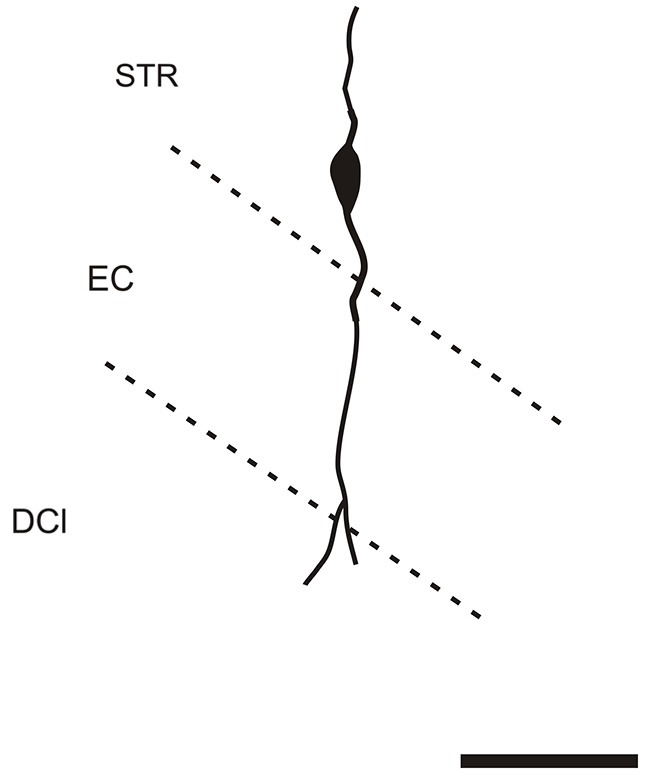
**Camera lucida drawing of striatal CR-ir neuron sending dendrite to the margin of the DCl**. EC—external capsule, DCl—dorsal part of the claustrum, STR—striatum. Scale 50 μm.

**Figure 9:**
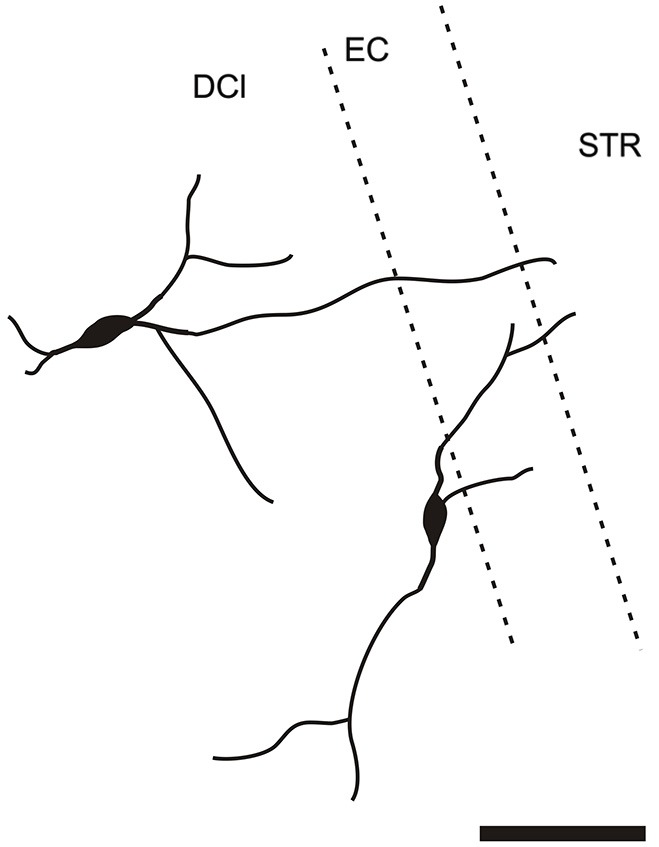
**Camera lucida drawing of two claustral CR-ir neurons sending dendrites to the margin of the striatum**. DCl—dorsal part of the claustrum, EC—external capsule, STR—striatum.

**Figure 10:**
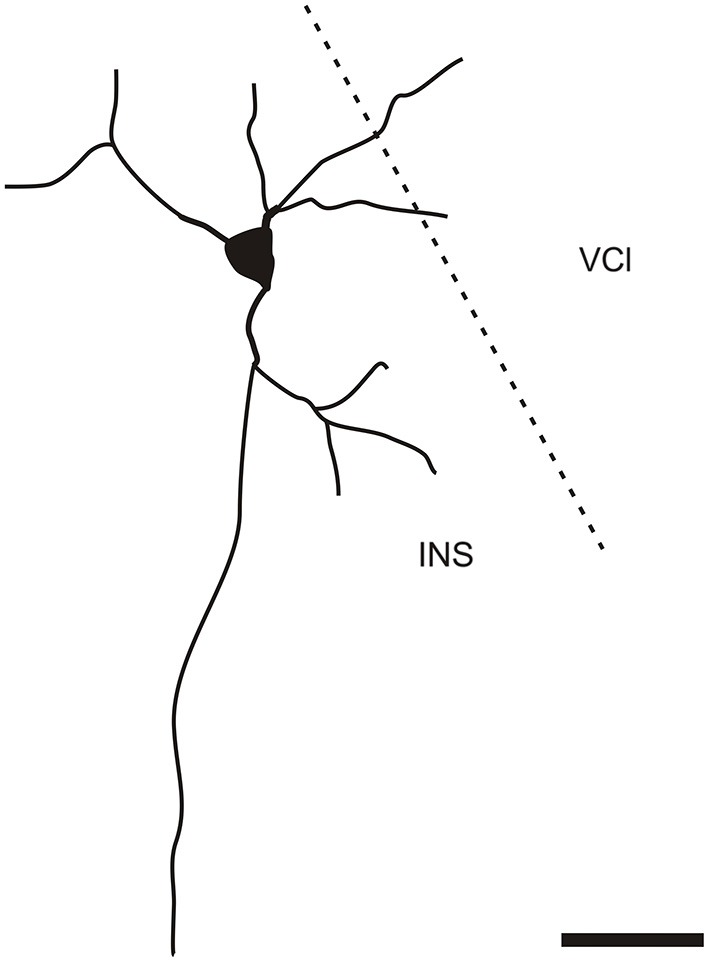
**Camera lucida drawing of CR-ir neuron located in the 6th layer of the insular cortex sending dendrites to the ventral part of the claustrum**. INS—insular cortex, VCl—ventral part of the claustrum. Scale 50 μm.

**Figure 11:**
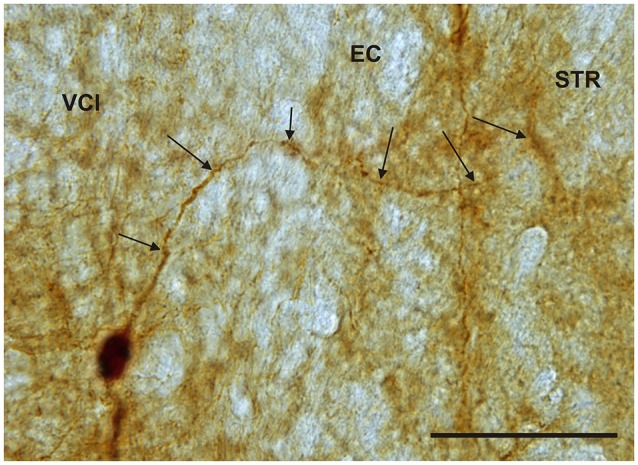
**Photomicrograph showing CR-ir neuron within the medial marginal part of the VCl sending dendrite (arrows) through external capsule (EC) to the marginal lateral part of the striatum (STR)**. Scale 50 μm.

**Figure 12:**
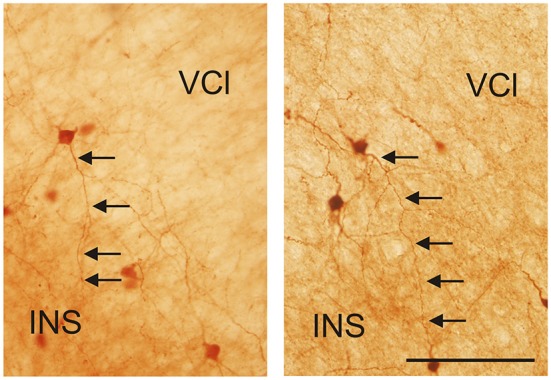
**Photomicrographs demonstrating CR-ir neurons located in the lateral marginal part of the VCl sending dendrites into 6th layer of the insular cortex (INS)**. Scale 100 μm.

It is of interest to note that a very small number of CR-ir striatal neurons displayed thin dendrites which traversed the external capsule and terminated in the medial marginal zone of the CLD (Figure 8). Further, in the CLD were seen dendrites branching from scattered CR-ir neurons within the medial and lateral marginal zones of the CLD, and then entering the adjoining marginal zone of the striatum (Figures 9, 11) as well as layer VI of agranular insular cortex (Figure 12). In addition small number of CR-ir neurons in layer VI of the insular cortex send processes (possibly dendrites) into the lateral marginal zone of the CLD (Figure 10).

#### Densitometry

A densitometric analysis was performed to quantify the observed differences in the CR-immunopositivity of neuropil between DCl, VCl, DEn, VEn and IEn. In all five subdivisions under study, the cROD was measured. The cROD values acquired in the DCl, VCl, DEn, VEn and IEn were then compared among each other. The results are summarized in Table 1. The highest cROD was observed in VEn (7.6 ± 1.0; dimensionless number ± standard error of mean, S.E.M.). The lowest density was measured in DCl (5.3 ± 0.4); however the difference between VEn and DCl did not reach statistical significance. To examine possible differences in the immunopositivity for CR along the rostrocaudal axis, we further compared the cROD between seven different rostrocaudal levels in examined areas. The results are summarized in Table 2. No significant differences in the cROD were found.

**Table 1:** **Corrected optical density ± S.E.M**.

DCl	VCl	DEn	IEn	VEn
5.3 ± 0.4	7.2 ± 0.5	5.4 ± 0.4	5.7 ± 1.0	7.6 ± 1.0

*Table 1—demonstrates corrected optical density in the subdivisions of the claustrum (DCl, VCl) and in the subdivisions of the endopiriform nucleus (DEn, IEn and VEn)*.

**Table 2:** **Corrected optical density ± S.E.M. at different rostrocaudal levels**.

	A	B	C	D	E	F	G
DCl	4.7 ± 0.7	5.7 ± 0.7	4.8 ± 1.1	5.4 ± 1.1	6.2 ± 1.3
VCl	6.6 ± 0.6	7.4 ± 1.0	5.8 ± 1.0	6.1 ± 1.1	7.4 ± 1.4
Den	5.7 ± 0.8	5.9 ± 0.9	4.6 ± 1.1	4.7 ± 0.8	5.7 ± 1.2	5.5 ± 1.8	6.2 ± 1.2
IEn	4.4 ± 0.9	5.7 ± 0.8	6.7 ± 2.0
VEn	7.0 ± 1.3	8.2 ± 1.8	7.5 ± 2.4

*Table 2—contains corrected optical density in sections at different rostrocaudal levels: section A (AP 2.1 mm), B (AP 1.2 mm), C (AP 0.2 mm), D (AP −0.8 mm), E (AP −1.8 mm), F (AP −2.8 mm) and G (AP −3.8 mm) relative to bregma (Paxinos and Watson, 2007)*.

## Discussion

This is the first study with detailed description of the distribution of CR-ir neurons in all subdivisions of the claustral complex in the rat.

To our knowledge, our findings are the first to demonstrate the existence of: (1) neurons in layer VI of the insular cortex sending processes (possibly dendrites) into the lateral marginal zone of the CLD, (2) CR-ir neurons in the CLD sending dendrites to the striatum; and (3) CR-ir neurons in the marginal area of the striatum sendings dendrites to the CLD.

Real et al. (2003) described moderate-to-dense immunoreactive neuropil throughout the DEn and VEn in the mouse, with a paucity of CR-ir neurons. The rat END consists of three subdivisions: the DEn, VEn and IEn. While the DEn and VEn shared pattern of labeling similar to the situation described in the mouse, the IEn displayed strongly positive medium-sized neurons, typically multipolar in shape.

If we compare CR-immunoreactivity in the claustrum of rodents such as the mouse and rat with other mammals, differences are clearly seen. There are fewer CR-ir neurons in the dorsal and ventral claustrum of the rabbit compared to the mouse (Real et al., 2003) and to the rat in the present study.

In the dorsal claustrum of the rabbit, CR-ir neurons were predominant in the medial half of the nucleus along with a moderate number of of thin positive fibers and puncta. Also, densely-stained fibers were noted along its medial border. The obvious subdivision of the dorsal claustrum into CR-positive and-negative sectors we report here in the rat is not described in the rabbit. It can also be said that CR-ir neurons in the ventral claustrum of the rabbit are extremely rare. CR-immunoreactivity in the claustrum of the cat (Rahman and Baizer, 2007) and monkey (Reynhout and Baizer, 1999) is comparable, with positive neurons homogenously distributed throughout the nucleus. While in monkey prevailed CR-ir neurons with bipolar morphology in the cat claustrum, in addition to elongated bipolar neurons also CR-positive neurons with large irregularly shaped somas were seen. Interestingly, the diffuse distribution of CR-positive neurons and fibers was also reported in the dorsal claustrum of the dolphin (Cozzi et al., 2014); the ventral claustrum was not described.

Structurally speaking, CR-ir neurons in the rabbit, cat and monkey claustrum are similar, with a preponderance of oval-shaped bipolar cells. In general, studies on rabbit and cat claustrum commonly report an abundance of multipolar neurons (Reynhout and Baizer, 1999; Wójcik et al., 2004; Rahman and Baizer, 2007). By way of comparison, the dolphin claustrum was reported to have small monopolar and bipolar neurons with round or fusiform cell bodies (Cozzi et al., 2014). With regard to functional considerations, the parcellation of the claustrum into several zones on the basis of reciprocal connectivity with sensory cortices suggests that it is a complex of modality-dependent units with upstream impact. This view is supported by the fact that majority of claustral cells are projection neurons, with but 7–12% comprised of GABAergic interneurons (Spahn and Braak, 1985). Based on corticoclaustral and claustrocortical projection patterns, studies indicate that CR-immunoreactivity in the claustrum of the rat and rabbit is not reflective of its functional organization (Sloniewski et al., 1986; Sadowski et al., 1997; Kowiański et al., 1998).

Immunohistochemical localization of the vesicular glutamate transporter (VGLUT2) in the mouse claustrum enables one to differentiate a VGLUT2-negative central core which corresponds to the VCl in the rat, and which is surrounded both dorsally and medially with a VGLUT2-positive plexus of fibers and puncta (Real et al., 2003). This VGLUT2-positive “shell” partly corresponds to the strongly CR-ir dorsal and medial marginal zones seen in the mouse and rat claustrum (Real et al., 2003; present results). Interestingly, the weakly CR-positive central core in the rat claustrum is strongly parvalbumin-positive and seen to contain a high density of parvalbumin-ir neurons, fibers and puncta (Druga et al., 1993). Additionally, projections from the ventroposterolateral parvicellular and ventroposteromedial parvicellular thalamic nuclei (which are considered to be relay nuclei for gustatory and visceral signals) terminate not only in the insular cortex but also in the dorsal and medial claustrum (Allen et al., 1991). This is seen as corresponding to the CR-positive zone in the DCl. Lastly, similar termination fields within the claustrum exhibited projections from the parabrachial nucleus (Allen et al., 1991).

Of particular note, our present data indicate that the dendrites of CR-ir neurons in the medial and lateral margins of the rat claustrum demonstrate the presence of direct reciprocal connectivity with neighboring structures, such as the striatum and insular cortex. This intriguing pattern of interconnectivity is consistent with the concept of a global processing and synchrony detection system, perhaps relative to the competitive actions of often disparate sensory afferents flowing into the claustrum (Smythies et al., 2012, 2014), and is in agreement with the proposal of some authors who suggested the importance of the claustrum for complex functions like perception, consciousness and cognitive processes (Crick and Koch, 2005).

## Conclusions

The calcium-binding protein CR was clearly and abundantly in evidence throughout the claustrum of the rat, comprised of immunopositive neurons, fibers and puncta. Both segments of the claustrum—the dorsal claustrum as well as all subdivisions of the END—contained CR-ir neurons, albeit with varying population densities. The majority of these positive neurons displayed small- or medium-sized round, oval or elongated cell bodies with aspiny dendrites, and were unevenly distributed. The DCl and the marginal parts of the VCl were noted to have the higher density of CR-ir neurons. In contrast, the central core of the VCl contained a paucity of CR-ir neurons with weakly immunoreactive neuropil. With respect to the END and its three subdivisions, CR-ir neurons were most prevalent in the IEn.

## Conflict of interest statement

The authors declare that the research was conducted in the absence of any commercial or financial relationships that could be construed as a potential conflict of interest.
